# Construct Validity of the SF-12v2 for the Homeless Population with Mental Illness: An Instrument to Measure Self-Reported Mental and Physical Health

**DOI:** 10.1371/journal.pone.0148856

**Published:** 2016-03-03

**Authors:** Antony Chum, Anna Skosireva, Juliana Tobon, Stephen Hwang

**Affiliations:** 1 School of Geography, University of Nottingham, Nottingham, United Kingdom; 2 Department of Psychiatry, University of Ottawa, Ottawa, Ontario, Canada; 3 Department of Psychiatry and Behavioural Neurosciences, McMaster University and Offord Center for Child Studies, Hamilton, Ontario, Canada; 4 St. Michael’s Hospital, Centre for Research on Inner City Health, Toronto, Ontario, Canada; University of Stellenbosch, SOUTH AFRICA

## Abstract

**Background:**

Self-reported health measures are important indicators used by clinicians and researchers for the evaluation of health interventions, outcome assessment of clinical studies, and identification of health needs to improve resource allocation. However, the application of self-reported health measures relies on developing reliable and valid instruments that are suitable across diverse populations. The main objective of this study is to evaluate the construct validity of the SF-12v.2, an instrument for measuring self-rated physical and mental health, for homeless adults with mental illness. Various interventions have been aimed at improving the health of homeless people with mental illness, and the development of valid instruments to evaluate these interventions is imperative.

**Study Design:**

We measured self-rated mental and physical health from a quota sample of 575 homeless people with mental illness using the SF-12v2, EQ-5D, Colorado Symptoms Index, and physical/mental health visual analogue scales. We examined the construct validity of the SF-12v2 through confirmatory factor analyses (CFA), and using ANOVA/correlation analyses to compare the SF-12v2 to the other instruments to ascertain discriminant/convergent validity.

**Results:**

Our CFA showed that the measurement properties of the original SF-12v2 model had a mediocre fit with our empirical data (χ^2^ = 193.6, df = 43, p < .0001, CFI = 0.85, NFI = 0.83, RMSEA = 0.08). We demonstrate that changes based on theoretical rationale and previous studies can significantly improve the model, achieving an excellent fit in our final model (χ^2^ = 160.6, df = 48, p < .0001, CFI = 0.95, NFI = 0.95, RMSEA = 0.06). Our CFA results suggest that an alternative scoring method based on the new model may optimize health status measurement of a homeless population. Despite these issues, convergent and discriminant validity of the SF-12v2 (scored based on the original model) was supported through multiple comparisons with other instruments.

**Conclusion:**

Our study demonstrates for the first time that the SF-12v2 is generally appropriate as a measure of physical and mental health status for a homeless population with mental illness.

## Introduction

Self-reported measures of health and wellbeing are important indicators for the purpose of monitoring and assessing functional health at individual and population levels. This information can be used by clinicians and researchers for the evaluation of health care [1], for assessing the outcome of clinical studies, for identifying health needs to improve resource allocation, and more generally for the comparison of health and wellbeing across different populations. However, the application of self-reported measures of health relies on the development of reliable and valid instruments that are suitable across diverse populations. The Short Form 12 Survey–version 2 (SF-12v2) [2], an abbreviated version of the Short Form 36 Survey [3], is an instrument for measuring self-rated physical and mental health through a 12-item questionnaire. See Table 1 for a full list of abbreviations used in this paper, and for information on the subscales and corresponding items measured on the SF-12v2. The SF-12v2 has demonstrated reliability and validity for measuring self-reported health status in the general population in the U.S. and other countries [3–5], and for clinical subgroups such as patients with a history of stroke [6], diabetes mellitus [7], and inflammatory-rheumatic disease [8]. Due to SF-12v2’s brevity, simplicity, and ease-of administration, the instrument may be particularly useful for researchers working with urban, hard-to-reach, and vulnerable populations. However, more work needs to be done to establish the reliability and validity of the instrument specific to these particularly vulnerable populations. The purpose of this study is to investigate the validity and reliability of the SF-12 v.2 for an urban, multiethnic, homeless population with mental illness since no previous studies have validated the instrument for this population.

**Table 1:** Abbreviations and description of SF-12v2 subdomains and items.

Abbreviation	Definition
**Instruments**	
SF-12v2	Short Form 12 Survey version 2
EQ-5D	Standardized instrument to measure health outcomes from EuroQol
CSI	Colorado Symptoms Index, a measure of mental illness symptoms
VAS	Visual Analogue Scale
**Analysis terms**	
CFA	Confirmatory Factor Analysis
CFI	Comparative Fit Index (measure of model fit for CFA)
RMSEA	Root Mean Square Error of Approximation (measure of model fit for CFA)
NFI	Normed Fit Index (measure of model fit for CFA)
TFL	Tucker-Lewis Index (measure of model fit for CFA)
PNFI	Parsimony Normed Fit Index (measure of model fit for CFA)
**SF-12v2 subdomains and items**	
PCS	Physical Component Summary of the SF-12v2
MCS	Mental Component Summary of the SF-12v2
PF	Physical Functioning subscale (2 items measured: moderate activities, and climbing several flights)
RP	Role Physical subscale (2 items: accomplished less, limited in kind)
RE	Role Emotional subscale (2 items: accomplished less, not careful)
GH	General Health subscale (1 item: rating of general health)
MH	Mental Health subscale (2 items: peaceful, downhearted/low)
SF	Social Functioning subscale (1 item: frequency health problems interfered with socializing)
BP	Bodily Pain subscale (1 item: pain interfere)
VT	Vitality subscale (1 item: energy)

In the North American and international context, various interventions have been developed and implemented with the aim of improving the health of homeless people with mental illness [9]. The development of reliable and valid instruments to evaluate these interventions is imperative. While the reliability and validity of SF-12 among people with severe mental illness has been investigated [10], https://docs.google.com/document/d/1Of5gRHLko6Uq7wzXOvaQev9rZft1V-20hfaZ_r3n75o/edit%20-%20heading=h.4d34og8 only one study has attempted to validate the SF-12 for the homeless population [11]. This study used the previous version of the instrument (i.e. SF-12, version 1), which contained different wording and questions than the most recent version (v.2). https://docs.google.com/document/d/1Of5gRHLko6Uq7wzXOvaQev9rZft1V-20hfaZ_r3n75o/edit-heading=h.4d34og8 In this United States (US) study, the author noted that some SF-12 items might not be appropriate for very poor and unemployed populations. For example, item content references daily activities such as “pushing a vacuum cleaner,” “bowling (or playing golf)” and evaluating whether the pain interferes with “normal work (including both work outside the home and housework).” The item content of SF-12 refers to the same daily activities as the preceding version of the instrument. As activity patterns vary qualitatively and quantitatively by socioeconomic class, the dearth of validity studies in homeless population is a concern. Despite the problematic item content references, the study demonstrated that the SF-12v1 differentiated between levels of severity of physical and mental health conditions and detected variation in health status. The study, however, did not explore the construct validity of the scale, was based on a small sample of homeless persons at a day shelter, and used the first version of the instrument.

The homeless population is different from the general population due to high physical and mental illness co-morbidity. People who are homeless are more likely to have poor mental health [12, 13], higher rates of substance abuse [14], chronic medical conditions [15, 16] and infectious diseases like tuberculosis, Hepatitis C, and HIV [17, 18], compared to the general population. Due to the differences in health status and other characteristics between people who are homeless and the general population, there is a critical need to explore the validity of the SF-12v2 before recommending it as a reliable and validated measure for use with homeless populations. The objective of this study is to address the existing knowledge gap by examining the construct validity (i.e. factorial, convergent, and discriminant validity) of the SF-12v2 for an urban, multiethnic, homeless population with mental illness (n = 574).

## Methods

### Data Collection

Data from this study is drawn from the At Home/Chez Soi project: a randomized controlled trial of a Housing First intervention. The intervention is aimed at meeting the housing and service needs of homeless people with mental illnesses, and details of this trial are described elsewhere [19, 20].

The target population is homeless adults with serious mental illness residing in Toronto, Canada. To be eligible for the study, participants had to 1) be 18 years of age or older, 2) be absolutely homeless (i.e., no fixed place to stay in the past 7 nights with little likelihood of finding a place in the next month) or precariously housed (i.e., living in a rooming house or hotel/motel as a primary residence AND have experienced 2 or more of episodes of being absolute homeless OR one episode of being absolutely homeless of at least 4 weeks duration in the past year, and 3) have a serious mental disorder. To establish the presence of a serious mental disorder, participants must have either had a history of recent psychiatric treatment, or were identified as having an eligible diagnosis identified by the DSM-IV criteria in the Mini International Neuropsychiatric Interview 6.0 [21]. Eligible diagnoses included 1) major depressive episode, 2) manic or hypomanic episode, 3) mood disorder with psychotic features, 4) panic disorder, 5) post-traumatic stress disorder, and 6) psychotic disorder.

While At Home/Chez Soi involved the randomization of participants into housing first and usual care study arms, the present paper analyzes the baseline data only, and participants from both treatment and usual care group are included in all analyses. Participants were recruited from October 2009 to June 2011 using a targeted recruitment and referral strategy. Referrals to the study came from Toronto’s extensive network of service providers for homeless and mentally ill individuals. A core group of more than 80 shelters, drop-in centres, hospitals, outreach programs, mental health services, and community health centers referred potential participants. In total, 1342 referrals were received from service providers, and 726 met the eligibility criteria (616 excluded due to reasons such as not absolutely homeless, lack of legal status in Canada, and being a current client of ACT or ICM treatment). At the screening interview, a further 151 persons were excluded (see Table 2 for detailed breakdown of reasons for exclusion). Targeted recruitment was used in the study to ensure representativeness of homeless individuals from specific demographic groups, and recruitment quotas were set based on a comprehensive 2006 census of homeless people [22]. Based on homeless census data, targeted recruitment was undertaken to ensure that approximately 25% of participants were women and approximately 40% of participants were from immigrant and ethno-racial groups. A quota was also set to recruit about 75% of participants at shelters, 17% from people living on the street, and 8% at health care facilities, prisons, and jails. A total of 575 individual (43% of all referrals) met all eligibility requirements, provided written informed consent, and completed screening and baseline interviews. The study was approved by the Research Ethics Board of St. Michael’s Hospital in Toronto, Canada, and was registered with the International Standard Randomized Control Trial Number Register (ISRCTN42520374).

**Table 2:** Reasons for exclusion at the screening interview (n = 151 excluded).

Reasons for exclusion	N
Did not meet serious mental disorder criteria	59
Declined consent	54
Was not absolutely homeless or precariously housed	16
Unable to provide informed consent	4
Lack of space in the relevant quota group	5
Current client of ACT or ICM treatment	9
Incomplete referral	1
Declined participation	1
Withdrew	1

### Measures

Generic self-rated mental and physical health from our sample were measured using the SF-12v2, the EuroQOL five dimensions questionnaire (EQ-5D) [23, 24], physical and mental health state visual analogue scales [25], and Colorado Symptoms Index (CSI) [26–29]. The latter two measures, described below, were used to assess the convergent and discriminant validity of the SF-12v2.

The SF-12v2 consists of 12 items, which are categorized into eight domains (or subscales): Bodily Pain (BP), General Health (GH), Vitality (VT), and Social Functioning (SF) with one item each; and physical Functioning (PF), Mental Health (MH), Role Physical (RP), and Role Emotional (RE) domains each with two items. To score the SF-12v2, we followed the method proposed by the original authors [30]. The summary scores of the SF-12v2, Physical Component Summary (PCS) and Mental Component Summary (MCS) scores, are calculated from z-scores of the 8 subscales, and all scales contribute to the scorings of PCS and MCS, using weights from principal component analysis on the SF-36 scales [31]. The norm-based scoring used here produces scores with a mean of 50 and a standard deviation of 10 for the US population; a higher score indicates better health [3, 32]. The EQ-5D is a self-administered standardized measure of health status developed by the EuroQoL Group and provides a simple, generic measure of health [23, 24]. It has five items measuring mobility, self-care, usual activities, pain/discomfort and anxiety/depression, each at three levels from level 1, with no problems, to level 3, which indicates a debilitating limitation in daily life for the specific dimension of health. The EQ-5D also features visual analog scales (EQ-5D VAS), on a 100-point scale from the “best imaginable health state” (100) to the “worst imaginable health state” (0). The EQ-5D has been shown to discriminate between severity subgroups of mental health patients and to capture improvements in health over time [33]. Furthermore, the EQ-5D has also been shown to discriminate chronic and mental disease status among the homeless population [34]. Two additional visual analogue scales, similar to the one found in EQ-5D, were also administered to measure the respondents’ physical health state and mental health state respectively (i.e. VAS-physical and VAS-mental). Previous studies have shown that single-item visual analogue scales measuring mental health had a 77% agreement with the 15-item depression scale of the Profile of Mood State [25], while visual analogue scale measuring physical health was 0.63 correlated with the Health Utility Index (HUI), and had a 0.76 correlation with a five-point rating of self-rated physical health [35]. These additional visual analogue scales were used to provide additional checks of construct validity in this study.

The Colorado Symptom Index (CSI) is a 14-item instrument that assesses the presence and frequency of mental illness symptoms experienced within the past month. It has proven to be a valid and reliable survey instrument for homeless individuals and those with mental health issues [26–29]. Responses are provided using a 5-point likert scale ranging from 0 (not at all) to 4 (at least every day). The item scores are summed, and a higher score indicates a higher level of symptoms.

### Statistical Analyses

Construct validity of the SF-12v2 was examined through confirmatory factor analysis (CFA) using structural equation modeling (SEM). CFA is a method used to test the hypothetical structure of a measure and to evaluate the fit of proposed measurement model for a given set of empirical data [36]. The major advantage of SEM for conducting CFA is that the validity of the expected factor structure can be evaluated by multiple goodness-of-fit indices [37]. Specifically, the comparative fit index (CFI), root mean square error of approximation (RMSEA), normed fit index (NFI), Tucker-Lewis index (TLI), and parsimony normed fit index (PNFI) were used for assessing the fit of the model to data [36]. The selection of cut-offs for the goodness-of-fit statistics were based on previous literature, i.e. a) for CFI and NFI, values of 0.95 and above is considered to represent a well-fitting model [38]; b) a RMSEA value of greater than 0.1 is considered to be poor fit, 0.08 to 0.1 is mediocre fit, and below 0.08 to be good/adequate fit [39]; c) a TLI value of 0.95 and above is considered to represent a well-fitting model [36]; d) no specific cut-offs are indicated for PNFI [40], but values closer to 1.00 represents a good fit and values closer to zero indicates a poor fit. Mulaik et al [40] notes that it is possible to obtain PNFI in the region of 0.5 even when the overall model fit is satisfactory.

In Ware et al’s [30] original model for the SF-12, the two-factor model most notably had uncorrelated physical and mental health factors with all items allowed to freely contribute to both factors in either a positive or negative way. Researchers have noted inconsistencies/problem in the instrument due to how all the items contribute to both factor. For example, Simon et al’s study of 536 primary care patients initiating antidepressant treatment [41] found that despite significant increases in Physical Fucntioning (PF), Role-Physical (RE), and the General Health Perceptions subscales over time (5 to 16 points, 95% CI) due to the treatment effect, the Physical Health Factor score remained unchanged due to the negative weighting of large positive changes (24–39 points, 95% CI) in Mental Health (MH), Social Functioning (SF), and the Role Emotional (RE) subscales. Some researchers since then have typically forced the scales for PF, RP, and BP to have zero loadings on the mental health factor, and simultaneously forced the MN, RE, and SF scales to have zero loadings on the physical health factor [6–8, 31]. CFA models now also commonly allow the physical and mental health factors to be correlated [6–8, 42, 43]. Evidence for allowing the mental and physical health factors to correlate come from two studies that found substantially better goodness-of-fit in the two-factor correlated model compared to models that forced the factors to be orthogonal [42, 43].

Based on these findings, we constructed our initial CFA model (model 1) based on Ware et al [30] with a) uncorrelated physical and mental health factors, and b) where all 12 items loaded onto both factors. For our model 2, we have correlated the mental and physical health factors, and items are forced to have zero loadings on their opposing health factors (based on specifications from previous studies [6–8]). In model 3, we build on model 2 by allowing correlated residuals for items from the same subscales on the basis that a) similarity in the wording of items that come from the same subscale, and b) these items would be expected to be more closely correlated with each other than items from other subscales. Correlated residuals for items from the same subscale have been used in two previous studies [8, 44]. Finally, in our last model tested (model 4), we also allowed the general health item (i.e. in general, would you say your health is: excellent, very good, good, fair, or poor) to load onto both the mental and physical health factor (rather than only physical health as in model 2 and 3).

Since the SF-12v2 items are measured with ordered-categorical responses, our CFA models used the weighted least squares estimation technique [45] with 4,000 bootstrapped samples, 95% CI, and significance tested with bias corrected confidence intervals for all factor loadings, intercepts, and variance. Analysis of moment structures (AMOS) 21 was used to evaluate how well the hypothesized models fit the observed data and to present the models statistically in diagram form.

To assess other components of construct validity, including convergent and discriminant validity [46], the associations between the SF-12v2 composite scores and other measures of participants’ physical and mental health were examined, including the EQ-5D, the Colorado Symptoms Index (CSI), and the visual analogue scales. The following hypotheses were tested:

1. Respondents who reported any health problems on the EQ-5D (i.e., levels 2 or 3) would have significantly more physical health and mental health problems as reported on the SF-12v2 (i.e., lower PCS and MCS) compared to those with no reported health problems.
2. The relationship between the SF-12v2 physical subscale (PCS) and the physical dimensions of the EQ-5D (i.e., mobility, self-care, usual activity, and pain/discomfort), and between the SF-12 mental subscale (MCS) and the mental dimension of EQ-5D (i.e., anxiety/depression), would be stronger (i.e., larger F-ratios) than between less comparable dimensions and composite scores.
3. Based on Cohen’s criteria [47], there would be a moderate to strong correlation (r ≥ 0.4), between the SF-12v2 physical subscale (PCS) and the visual analogue scale of physical health (VAS-physical), and between the SF-12v2 mental subscale (MCS) and the visual analogue scale of mental health (VAS-mental), and there would be a weak correlation (r ≤ 0.3) between PCS and VAS-mental, and between MCS and VAS-physical.
4. There would be a moderate to strong negative correlation (r ≥ 0.4) between the MCS and CSI, which measures severity of psychiatric symptomatology, and there would be a weak negative correlation (r ≤ 0.3) between the PCS and CSI.
5. The SF-12v2 items for a) limited in moderate activities and b) pain would have a significant (p<0.05) linear trend with VAS-physical, and the SF-12v2 item for c) health problems interfering with social functioning would have a significant linear trend (p<0.05) with VAS-mental.

Hypothesis 1 deals with known-groups validity, which is one component of construct validity. Hypotheses 1 and 2 (based on the relationships between the EQ-5D dimensions and SF-12v2 composite scores) were both analyzed using one-way analysis of variance (ANOVA). Hypotheses 3 and 4, (based on the relationships between SF-12v2 composite scores, CSI, and visual analogue scales) were examined using Pearson’s correlations. For the examination of both hypotheses, statistical test of difference between two dependent correlations will be used to determine whether the observed differences between the pairs of correlations are significant at the p<0.05 level [48]. For example, correlation between PCS and VAS-physical should be significantly different from the correlation between PCS and VAS-mental (in hypothesis 3), and the correlation between MCS and CSI should be significantly different from the correlation between PCS and CSI (in hypothesis 4). For hypothesis 5, our motivation for testing these three SF-12v2 items was due to their specific wording which might not be appropriate for a homeless, very poor, or unemployed population. Specifically, the items, a) “limitations in moderate activities” references pushing a vacuum cleaner, bowling, and playing golf, b) “pain” references limitations to housework and employment-based work, and c) “social functioning” references visiting friends and family, might not represent the everyday experiences of a poor homeless population due to the lack of a home, stable employment, and financial barriers. Examination of hypothesis 5 uses ANOVA contrast analysis to test for significant linear-trend between the aforementioned items and their related visual analogue scales. Two-tailed probability values under 0.05 were considered to be statistically significant. All statistical analyses were completed using SPSS v.21.

### Reliability

Cronbach's α coefficients were calculated to estimate the internal consistencies, or inter-item reliability, among items from SF-12v2 overall, the PCS subscale, and the MCS subscale in this study population. Based on the Nunnally criterion [49], Cronbach's α of .70 or greater was considered as a cut-off point for satisfactory internal consistency of the instrument.

## Results

The developers of the SF-12v2 recommend that a score is calculated only when responses are available for all items [50]. Thus, one person who did not answer all the SF-12v2 questions was removed, resulting in an analyzable sample of 574.

Table 3 summarizes the characteristics of participants for the sample overall, and displays the means and standard deviations for PCS, MCS, CSI, VAS-physical, and VAS-mental health scores cross-tabulated by socio-demographic characteristics. Study participants were 68.5% male, 54.4% Canadian-born, 95.8% single, 30% had been homeless for 1 to 3 years, and 31.9% were between the ages of 40–49 years old. Study participants were ethnically diverse with 58.7% who indicated one of the following ethnicities: Black (includes Black-African, Black-Caribbean, and Black-Canadian), East Asian, Indian-Caribbean, Latin American, Middle Eastern, South Asian, South-East Asian, and Mixed Ethnicity. This is not atypical given the high proportion of ethno-racial individuals in the general population in Toronto, at 49% [51], and previous studies have also indicated a high level of ethno-racial diversity in the Toronto homeless population [20, 52].

**Table 3:** Sample Characteristics, and the mean and standard deviations of SF-12 v.2 physical subscale, SF-12 v.2 mental subscale, CSI score, EQ-5D physical score, and EQ-5D mental score by demographic groups (n = 574).

	N (%)	Missing N (% of 574)	SF-12v2 Physical Health (PCS)	SF-12v2 Mental Health (MCS)	CSI Score (mental)	VAS Physical Health	VAS Mental Health
**Demographics**			Mean (SD)				
**Age**		0 (0.0)					
<30	138 (24.0)		49.9 (10.0)	34.7 (13.0)	39.9 (13.0)	66.0 (26.9)	58.9 (26.8)
30–39	133 (23.2)		47.4 (11.8)	33.9 (13.3)	41.4 (12.7)	62.1 (26.6)	50.4 (25.3)
40–49	183 (31.9)		45.0 (11.7)	34.0 (14.4)	41.1 (13.1)	62.4 (24.1)	55.0 (26.4)
≥50	120 (20.9)		42.2 (12.2)	38.8 (14.4)	36.2 (14.3)	58.5 (27.1)	59.2 (31.0)
p-value for ANOVA			**.000**	**.013**	**.011**	.393	.173
**Gender**		0 (0.0)					
Female	170 (29.6)		45.8 (12.2)	35.1 (14.0)	37.5 (12.6)	61.3 (28.3)	55.1 (27.8)
Male	393 (68.5)		46.3 (11.6)	35.3 (13.9)	40.7 (13.4)	62.9 (25.1)	56.3 (27.0)
Transgender/transexual	11 (1.9)		45.4 (12.4)	32.7 (14.7)	44.2 (17.9)	68.8 (24.4)	48.7 (32.7)
p-value for ANOVA			.877	.827	**.024**	.741	.756
**Country of Birth**		0 (0.0)					
Canada	312 (54.4)		46.4 (11.6)	33.9 (14.0)	40.8 (13.4)	61.5 (26.4)	53.5 (26.4)
Other	262 (45.6)		45.9 (11.9)	36.6 (13.7)	38.8 (13.2)	63.8 (25.6)	58.5 (28.2)
p-value for ANOVA			.613	**.019**	.081	.438	.102
**Ethnic or cultural identity**		0 (0.0)					
Non Ethno-racial	209 (36.4)		46.2 (11.7)	33.7 (14.3)	40.5 (13.8)	61.7 (25.7)	51.8 (26.2)
Ethno-racial	337 (58.7)		46.3 (11.9)	36.0 (13.7)	39.3 (13.3)	63.4 (26.5)	58.2 (27.8)
Aboriginal	28 (4.9)		44.0 (10.5)	35.4 (13.1)	42.2 (10.3)	56.2 (21.5)	49.7 (23.9)
p-value for ANOVA			.599	.177	.431	.592	.114
**Marital status**		3 (0.5)					
Married/cohabiting with partner	21 (3.7)		41.5 (14.0)	38.7 (15.9)	38.8 (15.3)	61.3 (30.6)	58.5 (30.6)
Single, divorced, widowed, never married	550 (95.8)		46.3 (11.6)	35.0 (13.8)	39.9 (13.3)	62.6 (25.9)	55.7 (27.2)
p-value for ANOVA			.063	.233	.711	.872	.740
**Length of homelessness**		13 (2.3)					
<1 year	127 (22.1)		46.4 (12.4)	34.8 (13.9)	37.5 (12.5)	65.7 (27.0)	57.6 (27.7)
≥1 to <3 years	138 (24.6)		47.3 (11.5)	35.5 (13.7)	39.4 (14.0)	65.5 (22.9)	56.3 (26.6)
≥3 to <7 years	150 (26.3)		46.1 (12.0)	34.4 (13.8)	41.1 (13.5)	59.8 (26.9)	54.5 (28.4)
≥7 years	146 (25.4)		44.5 (11.5)	35.2 (14.0)	41.6 (12.8)	56.7 (26.3)	52.7 (26.7)
p-value for ANOVA			.244	.929	.065	.083	.709
**Education history**		2 (0.3)					
<High school	278 (48.4)		45.2 (11.6)	35.3 (13.2)	40.5 (13.8)	57.0 (27.9)	53.7 (26.2)
Completed high school	108 (18.8)		49.4 (11.3)	34.1 (14.6)	39.9 (13.2)	70.1 (24.6)	60.5 (27.7)
Some post-secondary school	186 (32.4)		45.6 (12.0)	35.6 (14.6)	38.9 (12.8)	65.4 (22.4)	56.0 (28.5)
p-value for ANOVA			**.005**	.635	.451	**.001**	.240

One-way ANOVA was used to examine whether the mean health scores significantly varied across the categories within each socio-demographic characteristic. The PCS significantly varied across age groups and education levels (p<0.01), and the MCS significantly varied across age groups and country of birth (p<0.05). As expected, physical health (PCS) was negatively associated with age (p<0.01) and mental health (MCS) was best among older participants. Contrary to our expectations, the length of homelessness was not significantly associated with either MCS or PCS.

### Construct Validity

**Fig 1:**
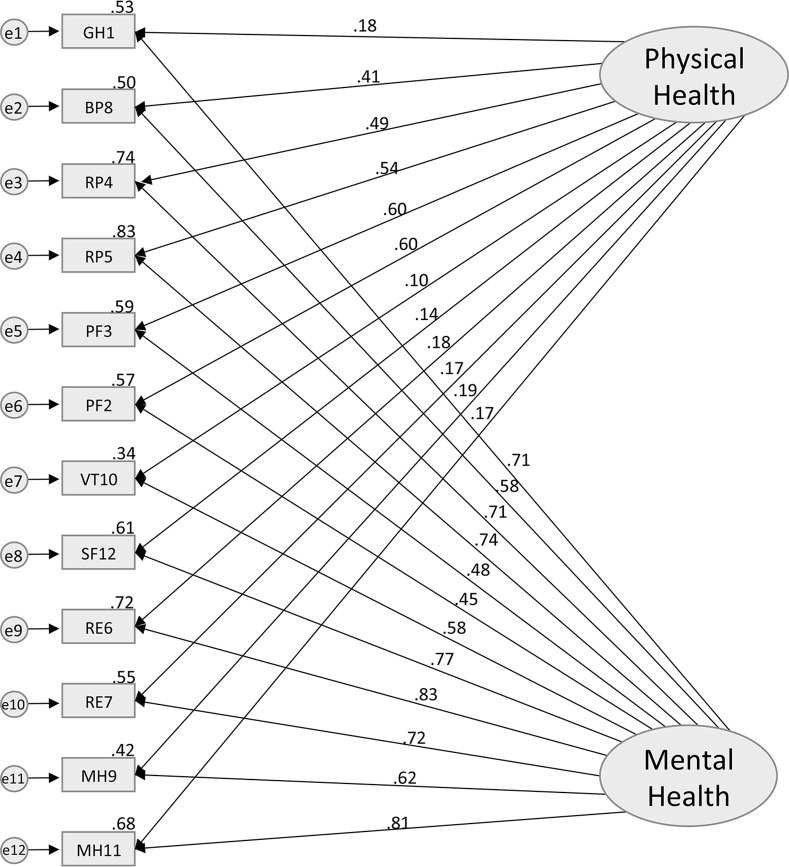
Standardized solution of the confirmatory factor analysis for the Model 1. GH1—General Health: rating of general health; PF2—Physical Functioning: moderate activities; PF3—Physical Functioning: climbing several flights of stairs; RP4—Role Functioning (Physical): accomplished less; RP5—Role Functioning (Physical): limited in the kind work or other activities; RE6—Role Functioning (Emotional): accomplished less (emotional problems); RE7—Role Functioning (Emotional): less carefully than usual; BP8—Bodily Pain: pain interference; MH9—Mental Health: calm and peaceful; VT10—Vitality: energy; MH11—Mental Health: downhearted and depressed; SF12—Social Functioning: health/emotional problems interfered with activities.

**Fig 2:**
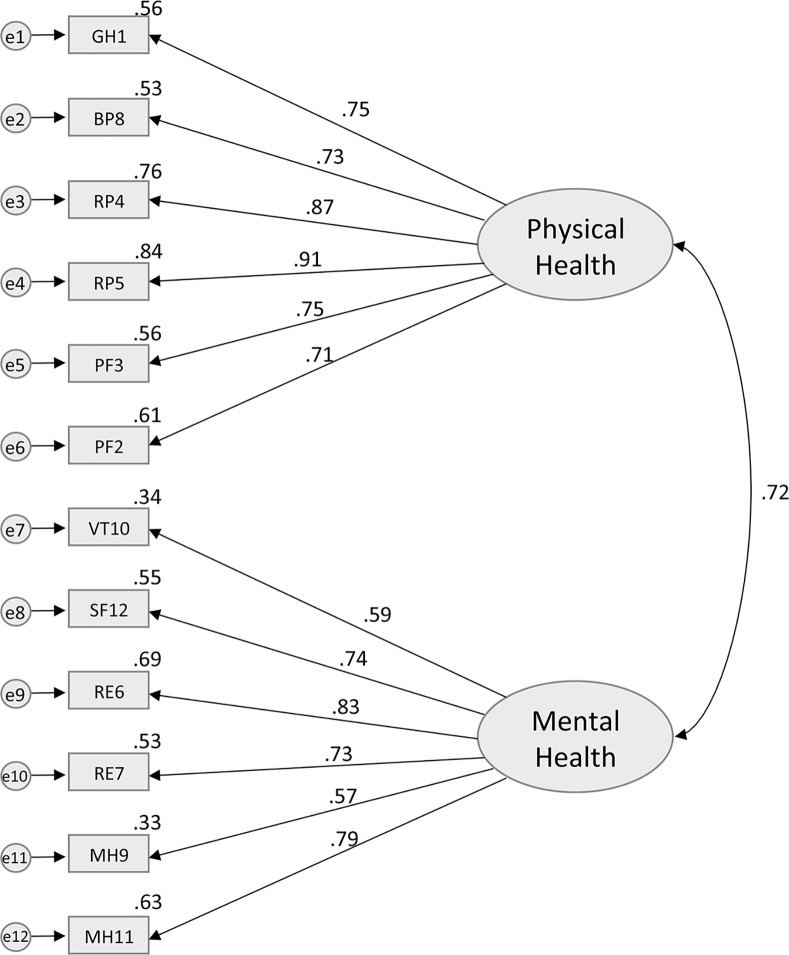
Standardized solution of the confirmatory factor analysis for the Model 2. GH1—General Health: rating of general health; PF2—Physical Functioning: moderate activities; PF3—Physical Functioning: climbing several flights of stairs; RP4—Role Functioning (Physical): accomplished less; RP5—Role Functioning (Physical): limited in the kind work or other activities; RE6—Role Functioning (Emotional): accomplished less (emotional problems); RE7—Role Functioning (Emotional): less carefully than usual; BP8—Bodily Pain: pain interference; MH9—Mental Health: calm and peaceful; VT10—Vitality: energy; MH11—Mental Health: downhearted and depressed; SF12—Social Functioning: health/emotional problems interfered with activities.

**Fig 3:**
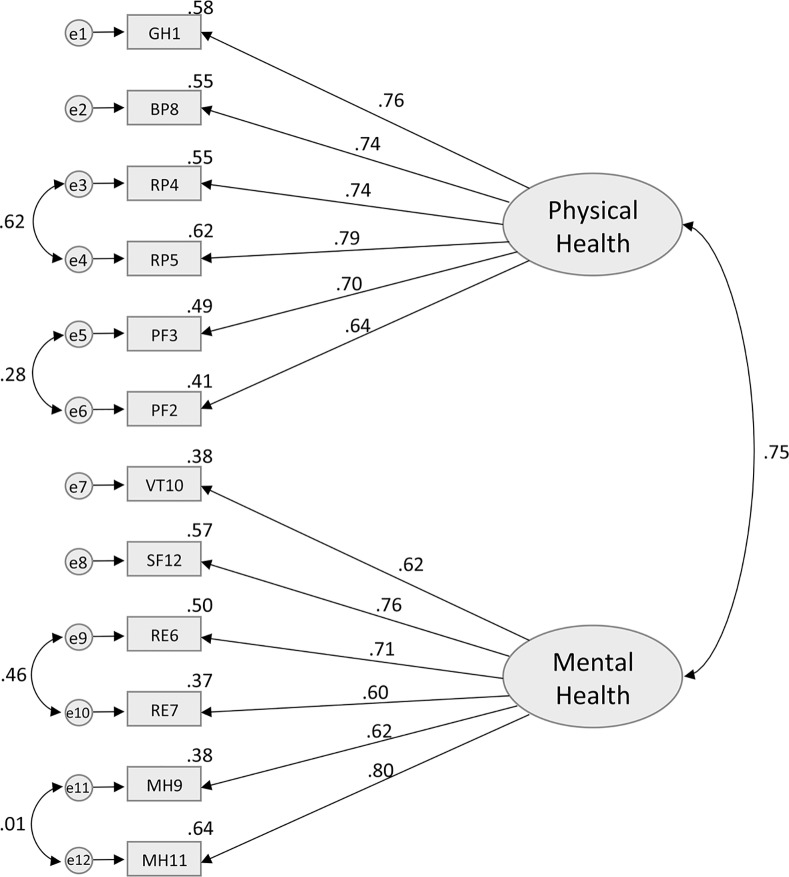
Standardized solution of the confirmatory factor analysis for the Model 3. GH1—General Health: rating of general health; PF2—Physical Functioning: moderate activities; PF3—Physical Functioning: climbing several flights of stairs; RP4—Role Functioning (Physical): accomplished less; RP5—Role Functioning (Physical): limited in the kind work or other activities; RE6—Role Functioning (Emotional): accomplished less (emotional problems); RE7—Role Functioning (Emotional): less carefully than usual; BP8—Bodily Pain: pain interference; MH9—Mental Health: calm and peaceful; VT10—Vitality: energy; MH11—Mental Health: downhearted and depressed; SF12—Social Functioning: health/emotional problems interfered with activities.

**Fig 4:**
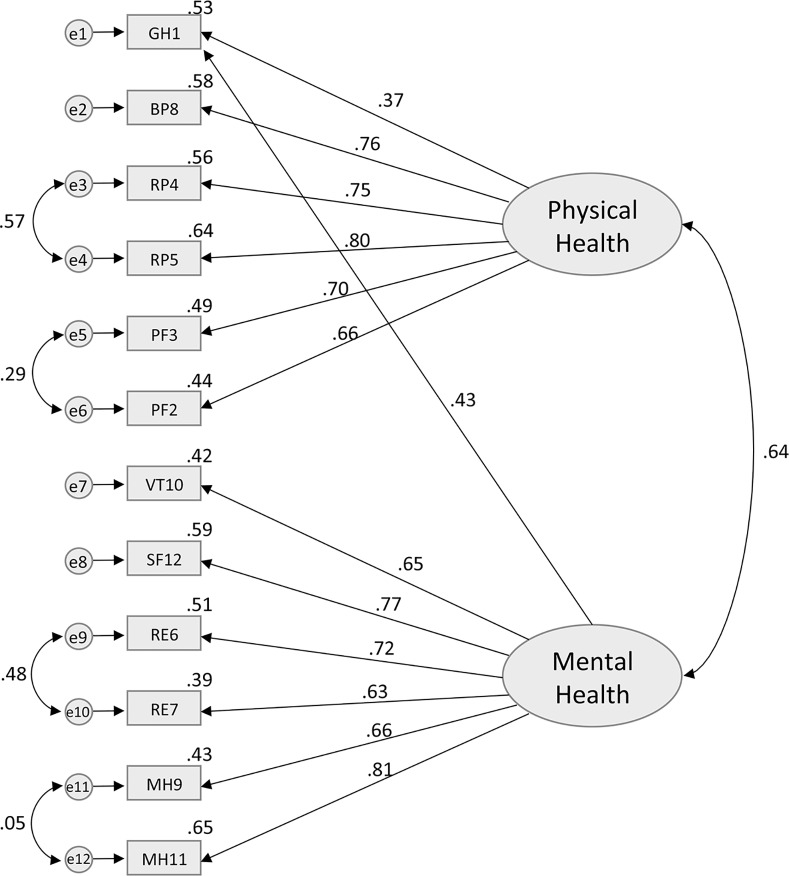
Standardized solution of the confirmatory factor analysis for the Model 4. GH1—General Health: rating of general health; PF2—Physical Functioning: moderate activities; PF3—Physical Functioning: climbing several flights of stairs; RP4—Role Functioning (Physical): accomplished less; RP5—Role Functioning (Physical): limited in the kind work or other activities; RE6—Role Functioning (Emotional): accomplished less (emotional problems); RE7—Role Functioning (Emotional): less carefully than usual; BP8—Bodily Pain: pain interference; MH9—Mental Health: calm and peaceful; VT10—Vitality: energy; MH11—Mental Health: downhearted and depressed; SF12—Social Functioning: health/emotional problems interfered with activities.

According to the original theoretical model of the SF-12 [3], Model 1 included two latent uncorrelated factors (PCS and MCS) each containing all 12 items presented in Fig 1. It has a χ^2^ of 193.6 (df = 43, p < .0001), with a CFI of 0.85, NFI of 0.83, and a RMSEA of 0.08 (95% C.I. = 0.07–0.09)–see Table 4. While both the CFI and NFI are below the preselected cut-off of 0.95 for a well-fitted model, the RMSEA value suggests that the model has a mediocre fit with the data. Model 2 (Fig 2), which added a correlation between the PCS and MCS factors, also showed an overall mediocre fit to the observed data (χ^2^ = 489.1, df = 53, p < .0001, CFI = 0.86, NFI = 0.84, RMSEA = 0.12). A moderate positive correlation of .61 (p < .0001), was found between PCS and MCS latent factors. While CFI and NFI remained similar for models 1 and 2, there was a notable decline in the model fit as indicated by the RMSEA. Model 3 (Fig 3) was specified with covariations between the error of the items that belong to the same subdomains (i.e., PF, RF, RE, and MH) in addition to having correlated latent factors. The analysis revealed that the model fit (χ^2^ = 204.3, df = 49, p < .0001, CFI = 0.95, NFI = 0.94, RMSEA = 0.07) was significantly better than Model 2. The RMSEA of 0.07 for the Model 3 suggested that some minor improvements in fit might still be possible, but overall an adequate to good fit was observed. Despite the good fit suggested by the RMSEA point estimate, it is important to note that the 95% CI of RMSEA (0.06–0.09) still includes values that are considered mediocre fit (i.e. >0.08–0.1). While CFI has reached the 0.95 cut-off representing a well-fitted model, NFI was just below the cut-off at 0.94, suggesting there may be room for improvement. In the final model (model 4), the GH item was allowed to cross-load onto MCS. Model 4 (Fig 4) had an excellent fit with the data (χ^2^ = 160.6, df = 48, p < .0001, CFI = 0.96, NFI = 0.95, RMSEA = 0.06), and the fit was an improvement upon model 3. There was further improvement in the point estimate of RMSEA from 0.07 to 0.06 (both defined as excellent fit); more importantly, the upper bound of the 95% CI for RMSEA shifted from 0.09 to 0.07, which is still considered to be excellent fit (i.e. RMSEA≤0.08). We also see further improvements in CFI and NFI, where both measures exceed the cut-off of 0.95 for a well-fitted model.

**Table 4:** Summary of fit statistics of the specified models (n = 574).

Fit indices	χ^2^; df; *p*	NFI	TLI	CFI	PNFI	RMSEA (95% CI)
**Model 1**	193.6; 43; .000	0.83	0.78	0.85	0.53	.08 (.07–.09)
**Model 2**	489.1; 53; .000	0.84	0.82	0.86	0.68	.12 (.11–.13)
**Model 3**	204.3; 49; .000	0.94	0.93	0.95	0.69	.07 (.06–.09)
**Model 4**	160.6; 48; .000	0.95	0.95	0.96	0.69	.06 (.06–.07)

NFI: Normed Fit Index; TLI: Tucker-Lewis Index; CFI: Comparative Fit Index; PNFI: Parsimony Normed Fit Index; RMSEA: Root Mean Square Error of Approximation.

While there are minor improvements in CFI, NFI, and RMSEA for model 4 (compared to model 3), we further investigated this using formal comparisons of the models. Since models 3 and 4 are nested models, we computed a chi-square test of difference to compare their relative goodness of fit. A significant change in the chi-square would suggest a substantial improvement in model fit. Comparison of model 3 (χ^2^ = 204.3) with model 4 (χ^2^ = 160.6), yields a difference in χ^2^ value of 43.7, a difference of 1 degree of freedom, and a p-value of <0.0001, suggesting model improvement. Akaike information criterion were also calculated for further model comparison [38, 49]. There are no standard cut-off values for a well fitted model, as it is a comparative measure of fit where lower value indicates better model fit. AIC values for models 3 and 4 were 233.895 and 183.293 respectively, which also suggests model improvement. Based on this set of evidence, Model 4 was selected as the best one of the group. Models 2, 3, and 4 showed a strong bivariate correlation between PCS and MCS (Figs 2, 3 and 4). It is important to note the improvement in the PNFI measure going from the uncorrelated factors model (model 1) to models with correlated latent factors (models 2, 3, and 4), which suggests that correlated factors and forced zero loadings have contributed to improved model parsimony.

While the chi-square statistic across all models described above were statistically significant, which may be an indicator of poor model fit, the chi-square statistic may be unreliable because it is influenced by having a large sample size or having more variables in the model (both conditions producing larger chi-squares) [36]. For this reason, other goodness-of-fit tests have been suggested (e.g. NFI, TFI, CFI, and RMSEA), and these are provided in our results.

### Convergent and discriminant validity

Convergent and discriminant validity were evaluated by comparing SF-12v2 results to the EQ-5D and CSI results. For the EQ-5D results, only five participants were at “level 3” (i.e., a debilitating limitation in daily life) for the mobility dimension and only three were level 3 for the self-care dimension. Due to low cell sizes, these responses were collapsed with level 2 for the mobility and self-care dimensions, respectively.

With the exception of the association between mobility levels and MCS (Table 5), each of the EQ-5D dimensions of health was significantly associated with PCS and MCS scores. With respect to hypothesis 1, participants who reported any problems in the EQ-5D health dimensions (i.e., levels 2 or 3) had significantly more self-reported physical health problems (lower PCS scores) and more mental health problems (lower MCS scores) compared to those who reported no problems (Table 5). However, the relationship between mobility and MCS was an exception to this pattern. There was no significant difference in the mean mental health sub-scores (MCS) between those with some mobility limitations and those without any mobility limitations.

**Table 5:** Mean (SD) SF-12v2 Composite Scores by EQ-5D Dimensions, and ANOVA results.

EQ-5D Dimensions	Level	n	PCS	F-ratio (p-value)	Eta^2^	MCS	F-ratio (p-value)	Eta^2^
Mobility*	1	343	51.42 (9.39)	**243.69 (.000)**	**29.88%**	35.95 (14.61)	2.79 (.095)	0.00%
	2	231	38.32 (10.50)			33.97 (12.73)		
Self-care*	1	450	48.00 (11.20)	**57.03 (.000)**	**9.07%**	35.98 (14.08)	**7.40 (.007)**	**1.28%**
	2	124	39.41 (11.30)			32.16 (12.86)		
Usual activities	1	343	49.14 (10.54)	**32.16 (.000)**	**10.12%**	38.13 (13.97)	**21.16 (.000)**	**6.86%**
	2	214	42.09 (11.98)			30.81 (12.43)		
	3	17	36.98 (12.83)			29.69 (14.73)		
Pain/discomfort	1	236	53.50 (8.22)	**139.82 (.000)**	**32.88%**	38.19 (15.76)	**11.23 (.000)**	**3.79%**
	2	240	43.32 (10.48)			33.81 (11.97)		
	3	98	35.37 (10.68)			31.13 (12.00)		
Anxiety/depression	1	137	50.84 (8.94)	**16.27 (.000)**	**5.39%**	47.04 (13.02)	**129.04 (.000)**	**31.13%**
	2	258	45.39 (11.89)			35.22 (11.18)		
	3	179	43.66 (12.48)			25.96 (10.89)		

*There were n = 5 and n = 3 participants who were level 3 (i.e., a debilitating limitation in daily life) for the mobility and self-care dimensions, respectively. Due to these low cell sizes, level 3 responses were collapsed with level 2 for the mobility and self-care dimensions.

Hypothesis 2 was confirmed by one-way ANOVA (Table 5). The relationships between the physical dimensions of the EQ-5D (i.e. mobility, self-care, usual activity, and pain/discomfort) and PCS were stronger than each dimensions’ relationship to MCS, confirmed by larger F-ratios. Also, as expected, the anxiety/depression EQ-5D dimension has a stronger association to MCS than to PCS.

For hypothesis 3, we examined the associations between SF-12v2 sub-scores and the visual analogue scales (VAS-physical and VAS-mental). PCS was positively correlated with VAS-physical at r = 0.56 (p<0.001), and with VAS-mental at r = 0.31 (p<0.001). The SF-12 mental sub-scale (MCS) was positively correlated with VAS-mental at r = 0.56 (p<0.001), and with VAS-physical at r = 0.34 (p<0.001). Using the statistical test of difference between two dependent correlations [48], we find that the both pairs of correlations (i.e. 0.56 vs. 0.31, and 0.34 vs. 0.56) are significantly different at the p<0.01 level. These correlations confirm hypothesis 3 that there would be moderate to strong correlations between matching scales (e.g. PCS and VAS physical health), and weak correlations between scales that measure different dimensions of health (e.g. PCS and VAS-mental health).

For hypothesis 4, CSI (lower scores represents fewer symptoms) was negatively associated with SF-12v2 scores (lower scores represent more symptoms): r = -0.264 for PCS (p<0.001) and r = -0.650 for MCS (p<0.001). The stronger correlation between MCS (mental subscale) and CSI than between PCS (physical subscale) and CSI confirms hypothesis 4 and provides evidence of convergent validity. Using the statistical test of difference between two dependent correlations [48], we find that the two correlations (i.e. -0.26 vs. -0.65) are significantly different at the p<0.01 level.

For hypothesis 5, SF-12v2 items for a) limitations to moderate activity and b) pain were compared to VAS-physical, and SF-12v2 item for c) social functioning was compared to VAS-mental. 1-way ANOVA and contrast test for linear trend results are presented in Table 6. We see that for all three items, the corresponding visual analogue scale increases/decreases systematically in the expected direction (i.e. VAS-physical decreases with each level increase of limitations to moderate activity and pain, and VAS-mental decreases with each level increase of social functioning limitation), and the test of linear trend for all three items is significant at p<0.001.

**Table 6:** SF-12 items by mean visual analogue scale score, ANOVA, and test of linear trend results.

**SF-12 items**	**Mean VAS-physical (Std. error)**	**N**	**ANOVA F-ratio (p-value)**	**Linear trend F-ratio (p-value)**
**Limitation to moderate activities**			50.01 (.000)	97.82 (.000)
Yes, limited a lot	43.82 (3.82)	70		
Yes, Limited a little	53.86 (2.65)	152		
No, not limited	69.43 (1.49)	352		
**Pain interfere with work**			30.88 (.000)	116.88 (.000)
Not at all	75.33 (1.87)	173		
A little bit	64.80 (2.45)	114		
Moderately	59.05 (2.99)	106		
Quite a bit	55.74 (2.97)	110		
Extremely	40.58 (4.10)	71		
	**Mean VAS-mental (Std. error)**	**N**	**F-ratio (ANOVA p-value)**	**Linear trend F-ratio (p-value)**
**Social functioning limitation**			46.95 (.000)	186.14 (.000)
All of the time	38.76 (3.08)	104		
Most of the time	44.93 (2.15)	138		
Some of the time	59.44 (2.62)	148		
A little of the time	67.26 (3.54)	62		
None of the time	76.25 (3.05)	122		

### Reliability

The Cronbach’s α for the SF-12v2 overall was 0.85, for the 6 items associated with physical subscale was 0.79, and for the 6 items associated with mental subscale was 0.79. These results all exceeded the Nunally threshold (i.e. >0.70) for internal consistency; however, these values alone may not be enough to demonstrate an adequate level of internal consistency. The authors of SF-12v2 recommend the analysis of test-retest reliability as a measure of internal consistency; however, this information was not available to our study and is therefore a limitation in this paper.

## Discussion

The objective of this study was to examine the construct validity of the SF-12v2 in an urban, multiethnic, homeless population with mental illness. The SEM analysis showed that the measurement properties of the original SF-12v2 model proposed by Ware et al. [30] only had a mediocre fit with our empirical data in the context of a two-factor measurement model with uncorrelated latent factors where all 12 item were cross-loaded onto both factors (i.e. model 1). Based on recent work on the factor structure of SF-12 described before, by adding in a) correlated latent factors, b) specific forced zero-loadings, c) covariation of errors in the items from the same subscale, and d) cross-loading of the GH item, we demonstrate that the original model for SF-12v2 can be significantly improved upon (i.e. model 4). Studies from the US and several European countries indicate that the GH item/subscale may cross-load to both components suggesting its mixed factor content [53, 54]. A strong correlation between the PCS and MCS measures (in models 2, 3, and 4) implies that physical and mental health should not be considered independently in the population of homeless people with mental illness. A strong correlation between the PCS and MCS measures was also shown in previous studies with various clinical populations [7, 8, 31]. This link between physical and mental health is not surprising, given that they share common risk factors. For example, sleep disorder is a core symptom of depression [55] and a risk factor for type II diabetes and cardiovascular diseases [56]. In a review of clinical populations [57], depressive symptoms is associated with increased poor health habits (e.g. smoking, over eating, and sedentary lifestyle), and increased morbidity and mortality from illnesses such as diabetes and heart disease. While these findings may have implications for SF-12v2 scoring and lead to other research questions regarding scoring methodology, e.g. comparing the validity of the standard scoring method verses scoring based on an alternative factor structure, this is beyond the scope of our study and similar work has been undertaken by other researchers [31]. We used the original scoring method to assess whether the application of SF-12v2 based upon an orthogonal scoring method could be recommended for a homeless population with mental illness, and also to maintain comparability with many older studies. However, we should note that our CFA models contribute to the growing evidence [6–8, 31, 42, 43] that suggests the scoring of SF-12v2 based on an oblique configuration may yield greater fit with empirical data compared to the standard proposed scoring based on an orthogonal configuration.

The convergent validity of the SF-12v2 scores was supported by moderate to strong associations between 1) the SF-12 physical health summary score (PCS) and the physical dimensions of the EQ-5D (i.e. mobility, self-care, usual activity, and pain/discomfort); 2) the PCS and the VAS-physical; 3) the SF-12 mental health summary score (MCS) and the VAS-mental health, and 4) the MCS and CSI score for mental illness symptoms. Discriminant validity was demonstrated through relatively weaker associations between the physical health scale of the SF-12v2 and mental scales of other measures and the mental health scale of the SF-12v2 and physical health scales of other instruments.

Given that some wording on the SF-12v2 referred to household chores, recreation, and visiting others (which may incur travel costs), we were concerned these items may not be appropriate for a homeless population. However, item level tests show significant linear trend between these items and their corresponding VAS (i.e. pain and moderate activities with the physical-VAS, and social functioning with the mental-VAS). This is evidence to support the ability for these items to predict physical and mental health in a homeless population with mental illness, despite our initial trepidation with the item wording.

While it was expected that respondents who reported any health problems on the EQ-5D would have significantly lower PCS and MCS scores for each dimension compared to those with no reported health problems, this was not the case for MCS and mobility. There were no significant differences in MCS scores across the 2 levels of mobility. These finding differed from a previous comparative analysis of the SF-12 and the EQ-5D for a general population in Canada [58], where both the PCS and MCS scores were consistently highest (i.e., better health and mental health) for groups who reported no problems (i.e. level 1) for all EQ-5D dimensions. However, as no previous comparisons between the SF-12 and EQ-5D exist for the homeless population with mental illness, the observed discrepancy may reflect a systematic difference between the study’s target population and the general population. Further research is needed to investigate this issue.

One limitation of this study is that our investigation did not include calculations of test-retest reliability, which is recommended by the authors’ of SF-12v2 [59]. While the SF-12v2 was re-administered in our randomized-controlled trial, the mean interval between the two tests was 6 months, and significant life-changes may have occurred in that period given the precarious nature of being homeless. Thus a decision was made to not calculate the test-retest reliability since any changes may be a result of changes in life circumstances rather than an indication of the extent that the instrument is able to produce stable and consistent results over time.

## Conclusions

While the results of this study generally support the construct validity of the SF-12v2 as a measure of generic physical and mental health status for an urban, multiethnic, homeless population with mental illness, it is important to point out certain caveats. The factor structure of the SF-12v2 original model (in the orthogonal configuration with 12 cross-loaded items) only had a mediocre fit with our empirical data, and can be improved to an excellent fit by specifying a) correlated latent factors, b) specific forced zero-loadings, c) covariation of errors in the items from the same subscale, and d) cross-loading of the GH item. The mediocre fit of the original model suggests that the standard scoring most commonly used may not be optimized to detect the level of generic physical and mental health status, and alternative scoring strategies should be considered to improve the performance of the SF-12v2 for homeless people with mental illness. Future research should examine the validity of the scores obtained through the standard method verses scores based on an alternative factor structure (e.g. obtained through CFA) for this population.

The SF-12 v.2 represents health status through a 12-item questionnaire, and its simplicity and ease of administration make the instrument an ideal measure for researchers working with urban, hard-to-reach, and vulnerable populations. Our study demonstrates for the first time that the SF-12 v.2 is generally an appropriate instrument to measure functional health and well-being of the homeless population with mental illness.

## References

[1] Kaplan RM. The significance of quality of life in health care. Quality of Life Research. 2003;12(1):3–16. 12803306 10.1023/a:1023547632545

[2] Ware JE, Sherbourne CD. The MOS 36-item short-form health survey (SF-36): I. Conceptual framework and item selection. Medical Care. 1992;30:473–48. 1593914

[3] Ware JE., Kosinski M, Keller SD. A 12-Item Short-Form Health Survey: construction of scales and preliminary tests of reliability and validity. Medical care. 1996;34(3):220–33. 8628042 10.1097/00005650-199603000-00003

[4] Gandek B, Ware JE, Aaronson NK, Apolone G, Bjorner JB, Brazier JE, et al Cross-validation of item selection and scoring for the SF-12 Health Survey in nine countries: results from the IQOLA Project. Journal of clinical epidemiology. 1998;51(11):1171–8. 9817135 10.1016/s0895-4356(98)00109-7

[5] Lundberg L, Johannesson M, Isacson DG, Borgquist L. The relationship between health-state utilities and the SF-12 in a general population. Medical Decision Making. 1999;19(2):128–40. 10231075 10.1177/0272989X9901900203

[6] Okonkwo OC, Roth DL, Pulley L, Howard G. Confirmatory factor analysis of the validity of the SF-12 for persons with and without a history of stroke. Quality of Life Research. 2010;19(9):1323–31. 10.1007/s11136-010-9691-8 20567914 PMC2952056

[7] Maurischat C, Herschbach P, Peters A, Bullinger M. Factorial validity of the Short Form 12 (SF-12) in patients with diabetes mellitus. Psychology Science. 2008;50(1):7.

[8] Maurischat C, Ehlebracht-König I, Kühn A, Bullinger M. Factorial validity and norm data comparison of the Short Form 12 in patients with inflammatory-rheumatic disease. Rheumatology international. 2006;26(7):614–21. 16179999 10.1007/s00296-005-0046-7

[9] Hwang SW, Tolomiczenko G, Kouyoumdjian FG, Garner RE. Interventions to improve the health of the homeless: a systematic review. American journal of preventive medicine. 2005;29(4):311-. e75. 16242595 10.1016/j.amepre.2005.06.017

[10] Salyers MP, Bosworth HB, Swanson JW, Lamb-Pagone J, Osher FC. Reliability and validity of the SF-12 health survey among people with severe mental illness. Medical Care. 2000;38(11):1141–50. 11078054 10.1097/00005650-200011000-00008

[11] Larson CO. Use of the SF‐12 Instrument for Measuring the Health of Homeless Persons. Health services research. 2002;37(3):733–50. 12132603 10.1111/1475-6773.00046 PMC1434659

[12] Public Health Agency of Canada. The human face of mental health and mental illness in Canada 2006. Ottawa: Minister of Public Works and Government Services Canada; 2006.

[13] Hwang SW. Homelessness and Health. Canadian Medical Association Journal. 2001;164(2):229–33. 11332321 PMC80688

[14] Goering P, Tolomiczenko G, Sheldon T, Boydell K, Wasylenki D. Characteristics of persons who are homeless for the first time. Psychiatric Services. 2002;53(11):1472–4. 12407279 10.1176/appi.ps.53.11.1472

[15] Hwang S, Dunn JR. Homeless People Galea S. & Vlahov D. (eds.), Handbook of Urban Health: Populations, Methods, and Practice, pp. 21–41. New York: Springer 2005.

[16] Acorn S. Mental and physical health of homeless persons who use emergency shelters in Vancouver. Hospital & community psychiatry. 1993;44:854–7. 8225298 10.1176/ps.44.9.854

[17] Khandor E, Mason K. The street health report 2007. Toronto, ON: Creative Commons; 2007.

[18] Beijer U, Wolf A, Fazel S. Prevalence of tuberculosis, hepatitis C virus, and HIV in homeless people: a systematic review and meta-analysis. The Lancet Infectious Diseases. 2012;12(11):859–70. 10.1016/S1473-3099(12)70177-9 22914343 PMC3494003

[19] Goering PN, Streiner DL, Adair C, Aubry T, Barker J, Distasio J, et al The At Home/Chez Soi trial protocol: a pragmatic, multi-site, randomised controlled trial of a Housing First intervention for homeless individuals with mental illness in five Canadian cities. BMJ open. 2011;1(2). 10.1136/bmjopen-2011-000323 PMC3221290 22102645

[20] Hwang SW, Stergiopoulos V, O’Campo P, Gozdzik A. Ending homelessness among people with mental illness: the At Home/Chez Soi randomized trial of a Housing First intervention in Toronto. BMC public health. 2012;12(1):787. 22978561 10.1186/1471-2458-12-787 PMC3538556

[21] Sheehan D, Lecrubier Y, Harnett Sheehan K, Janavs J, Weiller E, Keskiner A, et al The validity of the Mini International Neuropsychiatric Interview (MINI) according to the SCID-P and its reliability. European Psychiatry. 1997;12(5):232–41.

[22] City of Toronto Shelter Support and Housing Administration. Street Needs Assessment results. Toronto: City of Toronto; 2006.

[23] EuroQol G. EuroQol—a new facility for the measurement of health-related quality of life. Health policy (Amsterdam, Netherlands). 1990;16(3):199. 10.1016/0168-8510(90)90421-9 10109801

[24] Brooks R. EuroQol: the current state of play. Health policy. 1996;37(1):53–72. 10158943 10.1016/0168-8510(96)00822-6

[25] Killgore W. The visual analogue mood scale: can a single-item scale accurately classify depressive mood state? Psychological reports. 1999;85(3f):1238–43. 10710979 10.2466/pr0.1999.85.3f.1238

[26] Selim AJ, Rogers W, Fleishman JA, Qian SX, Fincke BG, Rothendler JA, et al Updated US population standard for the Veterans RAND 12-item Health Survey (VR-12). Quality of Life Research. 2009;18(1):43–52. 10.1007/s11136-008-9418-2 19051059

[27] Boothroyd RA, Chen HJ. The psychometric properties of the Colorado Symptom Index. Administration and Policy in Mental Health and Mental Health Services Research. 2008;35(5):370–8. 10.1007/s10488-008-0179-6 18561020

[28] Conrad KJ, Yagelka JR, Matters MD, Rich AR, Williams V, Buchanan M. Reliability and validity of a modified Colorado Symptom Index in a national homeless sample. Mental Health Services Research. 2001;3(3):141–53. 11718206 10.1023/a:1011571531303

[29] Greenwood RM, Schaefer-McDaniel NJ, Winkel G, Tsemberis SJ. Decreasing psychiatric symptoms by increasing choice in services for adults with histories of homelessness. American journal of community psychology. 2005;36(3–4):223–38. 16389497 10.1007/s10464-005-8617-z

[30] Ware JE, Kosinski M, Keller SD. A 12-Item Short-Form Health Survey: construction of scales and preliminary tests of reliability and validity. Medical care. 1996;34(3):220–33. 8628042 10.1097/00005650-199603000-00003

[31] Fleishman JA, Selim AJ, Kazis LE. Deriving SF-12v2 physical and mental health summary scores: a comparison of different scoring algorithms. Quality of Life Research. 2010;19(2):231–41. 10.1007/s11136-009-9582-z 20094805

[32] Ware JE, Kosinski M, Turner-Bowker DM, Gandek B. How to score version 2 of the SF-12 health survey (with a supplement documenting version 1) Lincoln, RI: QualityMetric Incorporated; 2002.

[33] Lamers L, Bouwmans C, Van Straten A, Donker M, Hakkaart L. Comparison of EQ‐5D and SF‐6D utilities in mental health patients. Health economics. 2006;15(11):1229–36. 16625671 10.1002/hec.1125

[34] Sun S, Irestig R, Burström B, Beijer U, Burström K. Health-related quality of life (EQ-5D) among homeless persons compared to a general population sample in Stockholm County, 2006. Scandinavian journal of public health. 2012;40(2):115–25. 10.1177/1403494811435493 22327187

[35] Belanger A, Berthelot J, Guimond E, Houle C. A head-to-head comparison of two generic health status measures in the household population: McMaster Health Utilities Index (mark 3) and the EQ-5D Ottawa: Statistics Canada, Health Analysis and Modelling Group 2000.

[36] Byrne BM. Structural equation modeling with AMOS: Basic concepts, applications, and programming: CRC Press; 2009.

[37] Quintana SM, Maxwell SE. Implications of recent developments in structural equation modeling for counseling psychology. The Counseling Psychologist. 1999;27(4):485–527.

[38] Hu Lt, Bentler PM. Cutoff criteria for fit indexes in covariance structure analysis: Conventional criteria versus new alternatives. Structural equation modeling: a multidisciplinary journal. 1999;6(1):1–55.

[39] MacCallum RC, Browne MW, Sugawara HM. Power analysis and determination of sample size for covariance structure modeling. Psychological methods. 1996;1(2):130.

[40] Mulaik SA, James LR, Van Alstine J, Bennett N, Lind S, Stilwell CD. Evaluation of goodness-of-fit indices for structural equation models. Psychological bulletin. 1989;105(3):430.

[41] Simon GE, Revicki DA, Grothaus L, Vonkorff M. SF-36 summary scores: are physical and mental health truly distinct. Medical care. 1998;36(4):567–72. 9544596 10.1097/00005650-199804000-00012

[42] Anagnostopoulos F, Niakas D, Tountas Y. Comparison between exploratory factor-analytic and SEM-based approaches to constructing SF-36 summary scores. Quality of Life Research. 2009;18(1):53–63. 10.1007/s11136-008-9423-5 19034689

[43] Hann M, Reeves D. The SF-36 scales are not accurately summarised by independent physical and mental component scores. Quality of Life Research. 2008;17(3):413–23. 10.1007/s11136-008-9310-0 18259888

[44] Wilson D, Tucker G, Chittleborough C. Rethinking and rescoring the SF-12. Sozial-und Präventivmedizin. 2002;47(3):172–7. 12238299 10.1007/BF01591889

[45] Finney SJ, DiStefano C. Non-normal and categorical data in structural equation modeling. Structural equation modeling: A second course. 2006:269–314.

[46] Mokkink LB, Terwee CB, Knol DL, Stratford PW, Alonso J, Patrick DL, et al The COSMIN checklist for evaluating the methodological quality of studies on measurement properties: a clarification of its content. BMC medical research methodology. 2010;10(1):22. 20298572 10.1186/1471-2288-10-22 PMC2848183

[47] Cohen J. Statistical Power Analysis for the Behavioral Sciences Second ed. Hillsdale, NJ: Lawrence Erlbaum Associates; 1988.

[48] Lee IA, Preacher KJ. Calculation for the test of the difference between two dependent correlations with one variable in common. 2013.

[49] Nunnally J, Bernstein I. Psychometric Theory. 3rd ed. New York: McGraw-Hill; 1994.

[50] Ware JE, Kosinski M, Turner-Bowker DM, Sundaram M, Gandek B, Maruish ME. User's Manual for the SF-12v2 Health Survey Second Edition: QualityMetric, Incorporated; 2009.

[51] Statistics Canada. National Household Survey: Profile files, 2011 Ottawa, Canada: Government of Canada; 2011 [cited 2014 July 10]. Available from: http://www12.statcan.gc.ca/nhs-enm/2011/dp-pd/prof/index.cfm?Lang=E.

[52] Khandor E, Mason K, Chambers C, Rossiter K, Cowan L, Hwang SW. Access to primary health care among homeless adults in Toronto, Canada: results from the Street Health survey. Open Medicine. 2011;5(2):e94 21915240 PMC3148004

[53] Ware JE. SF36 Health Survey Update. Spine. 2000;25(24):3130–9. 11124729 10.1097/00007632-200012150-00008

[54] Ware JE Jr, Kosinski M, Gandek B, Aaronson NK, Apolone G, Bech P, et al The factor structure of the SF-36 Health Survey in 10 countries: results from the IQOLA Project. Journal of clinical epidemiology. 1998;51(11):1159–65. 9817133 10.1016/s0895-4356(98)00107-3

[55] Nutt D, Wilson S, Paterson L. Sleep disorders as core symptoms of depression. Dialogues in Clinical Neuroscience. 2008;10(3):329–36. 18979946 10.31887/DCNS.2008.10.3/dnutt PMC3181883

[56] Touma C, Pannain S. Does lack of sleep cause diabetes? Cleveland clinic journal of medicine. 2011;78(8):549–58. 10.3949/ccjm.78a.10165 21807927

[57] Katon WJ. Clinical and health services relationships between major depression, depressive symptoms, and general medical illness. Biological psychiatry. 2003;54(3):216–26. 12893098 10.1016/s0006-3223(03)00273-7

[58] Johnson JA, Pickard AS. Comparison of the EQ-5D and SF-12 health surveys in a general population survey in Alberta, Canada. Medical care. 2000;38(1):115–21. 10630726 10.1097/00005650-200001000-00013

[59] Ware J, Kosinski M, Turner-Bowker DM, Gandek B. User’s manual for the SF-12v2 Health Survey. Lincoln, RI: QualityMetric Incorporated 2002.

